# Application of high-frequency jet ventilation for patients with severe traumatic brain injury

**DOI:** 10.1186/cc9730

**Published:** 2011-03-11

**Authors:** DM Sabirov, RN Akalaev, MB Krasnenkova, AL Rosstalnaya

**Affiliations:** 1 Tashkent Institute of Postgraduate Medical Education, Tashkent, Uzbekistan

## Introduction

We carried out research of a brain blood-groove with the purpose of estimating cerebrovascular effects with high-frequency artificial ventilation of lungs in 30 patients with severe traumatic brain injury.

## Methods

Traditional intensive therapy in conditions of various modes of respiratory support was performed: CMV - 10 patients, SIMV - 10 patients, HFJV - 10 patients. Adequacy of modes of ventilation was estimated on SpaO_2 _96 to 99%, and pCO_2 _34.7 to 35.2 mmHg. The median age was 36 ± 6 years, GCS was 7 to 9 points; the level of ICP exceeded 15 mmHg. We registered the cerebral blood flow velocity (Vm), resistance pial vessels (Pi), and dilatation reserve (Ri).

## Results

The analysis of parameters of central and system hemodynamics with varying respiratory support revealed significant distinctions. At mode CMV: ICP - 28.6 ± 0.7 mmHg; Vm - 51.1 ± 1.4 cm/second; Pi - 1.84 ± 0.1; Ri - 1.28 ± 0.01; CPP - 67.4 ± 1.3 mmHg. At SIMV: ICP - 31.7 ± 1.7 mmHg; Vm - 52.6 ± 4.1 cm/second; Pi - 1.60 ± 0.1; Ri - 1.23 ± 0.02; CPP - 68.0 ± 2.8 mmHg. At HFJV: ICP - 18.8 ± 2.9 mmHg; Vm - 57.8 ± 7.1 cm/second; Pi - 1.39 ± 0.2; Ri - 1.36 ± 0.01; CPP - 64.1 ± 6.1 mmHg. At CMV adverse conditions for venous return that can be accompanied by depression of intimate emission are created. Decrease in intimate emission will lead to decreased CPP that leads to spasm of pial vessels, and the dilatation reserve will not react to increased tone of pial vessels. At variance, SIMV is markedly similar to CMV interference of autoregulation parameters of the brain blood-groove and system hemodynamics. At HFJV there are no negative phenomena inherent in traditional ventilation. Presence of the kept or increased intimate emission appears to provide more chance to keep cerebral perfusion. At HFJV an authentically lower level of resistance Pi, higher parameter of Ri and lower ICP is marked. This interferes with occurrence of the expressed spasm and ischemia of the brain. At both variants of traditional ALV, the expressed infringements of perfusion and resistance of vessels of the pial-capillary system accompanied by substantial growth are marked.

## Conclusions

HFJV as respiratory support in severe traumatic brain injury, on a background of intracranial hypertension, has doubtless advantages before traditional methods of ALV. Its application provides preservation of active autoregulation of brain blood circulation, and promotes stabilization of intracranial pressure at a lower level.

